# pT3 colorectal cancer revisited: a multicentric study on the histological depth of invasion in more than 1000 pT3 carcinomas—proposal for a new pT3a/pT3b subclassification

**DOI:** 10.1038/s41416-022-01889-1

**Published:** 2022-07-21

**Authors:** Sebastian Foersch, Corinna Lang-Schwarz, Markus Eckstein, Carol Geppert, Maxime Schmitt, Björn Konukiewitz, Tanja Groll, Felix Schicktanz, Jutta Engel, Moritz Gleitsmann, Christina C. Westhoff, Nadine Frickel, Anne-Sophie Litmeyer, Albert Grass, Paul Jank, Sebastian Lange, Markus Tschurtschenthaler, Dirk Wilhelm, Wilfried Roth, Michael Vieth, Carsten Denkert, Iris Nagtegaal, Wilko Weichert, Moritz Jesinghaus

**Affiliations:** 1 Institute of Pathology, University Medical Center, Mainz, Germany; 2 Institute of Pathology, Friedrich-Alexander-University Erlangen-Nuremberg, Klinikum Bayreuth, Bayreuth, Germany; 3 Institute of Pathology, University Hospital Erlangen, Erlangen, Germany; 4 Institute of Pathology, Technical University of Munich, Munich, Germany; 5 Institute of Pathology, Philipps University Marburg and University Hospital Marburg (UKGM), Marburg, Germany; 6 Institute of Pathology, Christian-Albrechts University, Kiel, Germany; 7 Munich Cancer Registry (MCR), Institute for Medical Information Processing, Biometry, and Epidemiology (IBE), Ludwig-Maximilian-University (LMU), Munich, Germany; 8 II Medizinische Klinik, Klinikum rechts der Isar, Technical University Munich, Munich, Germany; 9 Institute for Translational Cancer Research, German Cancer Consortium (DKTK), Partner Site Munich, Munich, Germany; 10 Department of Surgery, Klinikum rechts der Isar, Technical University Munich, Munich, Germany; 11 Department of Pathology, Radboudumc, Nijmegen, The Netherlands; 12 German Cancer Consortium (DKTK), Partner Site Munich, Munich, Germany; 13 Bavarian Cancer Center (BZKF), Munich, Germany; 14 Comprehensive Cancer Center Munich (CCCM), Munich, Germany

**Keywords:** Colorectal cancer, Colorectal cancer

## Abstract

**Background:**

Pathological TNM staging (pTNM) is the strongest prognosticator in colorectal carcinoma (CRC) and the foundation of its post-operative clinical management. Tumours that invade pericolic/perirectal adipose tissue generally fall into the pT3 category without further subdivision.

**Methods:**

The histological depth of invasion into the pericolic/perirectal fat was digitally and conventionally measured in a training cohort of 950 CRCs (Munich). We biostatistically calculated the optimal cut-off to stratify pT3 CRCs into novel pT3a (≤3 mm)/pT3b (>3 mm) subgroups, which were then validated in two independent cohorts (447 CRCs, Bayreuth/542 CRCs, Mainz).

**Results:**

Compared to pT3a tumours, pT3b CRCs showed significantly worse disease-specific survival, including in pN0 vs pN+ and colonic vs. rectal cancers (DSS: *P* < 0.001, respectively, pooled analysis of all cohorts). Furthermore, the pT3a/pT3b subclassification remained an independent predictor of survival in multivariate analyses (e.g. DSS: *P* < 0.001, hazard ratio: 4.41 for pT3b, pooled analysis of all cohorts). While pT2/pT3a CRCs showed similar survival characteristics, pT3b cancers remained a distinct subgroup with dismal survival.

**Discussion:**

The delineation of pT3a/pT3b subcategories of CRC based on the histological depth of adipose tissue invasion adds valuable prognostic information to the current pT3 classification and implementation into current staging practices of CRC should be considered.

## Introduction

Colorectal carcinoma (CRC) is the third most common cancer in humans concerning incidence and mortality worldwide [1, 2]. Pathological pTNM staging according to the guidelines of the Union for International Cancer Control (UICC) [3] is the most robust prognostic factor in CRC and builds the backbone of post-operative clinical decision-making. The combined UICC stage summarises the different components of the TNM classification (pT: the extent of local invasion, pN: regional nodal status, pM: presence/absence of distant metastases) into a 4-tiered staging system (UICC I–IV), based on which CRC patients generally receive stage-adapted treatment. According to current guidelines [4–6], UICC Stage III patients (Any pT, pN+, c/pM0) are generally intended to receive adjuvant chemotherapy. Post-operative chemotherapy for UICC Stage II patients (pT3, pT4, pN0, c/pM0) is optional and currently only considered for Stage II patients that fulfil the criteria of a so-called “high-risk” profile (e.g. pT4, WHO high-grade CRC, insufficient lymph nodes sampled).

pT staging of CRC currently comprises four main categories ranging from pT1 to pT4a/b. CRCs that invade the pericolic/perirectal adipose tissue are classified as pT3, without further subdivision [3]. Therefore, the current pT3 category naturally comprises a wide range of CRCs ranging from cancers where only a few isolated tumour cells superficially infiltrate the adipose tissue to those that show an extensive and very deep infiltration of the fat tissue. This regularly includes pT3 tumours, where a perforation of the visceral peritoneum, and thus upstaging to the pT4a category, is only prevented by a very thin layer of connective tissue.

Although it is conceivable, that the clinical course between this wide range of CRCs summarised in the current pT3 category might be considerably variable, the extent of adipose tissue infiltration is not reflected in the current pathological staging practice of CRC.

Our study aimed to investigate, whether the current single-tier pT3 classification constitutes the optimal local staging scheme for adipose tissue invasive CRCs or if a subclassification of the pT3 category based on depth/extent of adipose tissue infiltration adds valuable prognostic information that might impact the clinical management of CRC. To this end, using digital pathology and biostatistical analyses, we investigated the histological depth of invasion into the pericolic/perirectal fat in three independent CRC cohorts from three maximum medical care hospitals in Germany (1939 CRCs) and correlated the results with disease-specific survival (DSS, including in pN/pM subgroups) in uni- and multivariate statistical analyses. Furthermore, we investigated the concordance of our digital measurements with a conventional measurement approach using classical light microscopy, in order to ensure the transferability of our approach into all settings of daily routine pathology.

## Patients and methods

### Training cohort and validation cohorts

A total of 1939 CRC patients (all pT stages) undergoing surgical resection between 1997 and 2019 from three maximum medical care hospitals in Germany (University Hospital Klinkum rechts der Isar, Munich; University Hospital Mainz, Germany; Hospital Bayreuth, Germany) were included in this study. The training cohort (cohort 1) consisted of 950 CRCs from the University Hospital Klinikum rechts der Isar of the Technical University of Munich, Germany, the two validation cohorts consisted of 542 (University Hospital Mainz, Germany, validation cohort 1) and 447 CRCs (Klinikum Bayreuth, validation cohort 2). Other neoplasms of the colorectal system than CRC (e.g. neuroendocrine tumours, non-epithelial tumours etc.), appendiceal tumours as well as cases with incomplete clinicopathological/survival data or insufficient tissue were excluded. Survival data, as well as clinicopathological characteristics from all patients, were extracted from local cancer registries or from hospital records. As described previously [7], events for DSS were defined as patient deaths that were clearly attributed to a progressive tumour disease by the treating clinician, while overall survival (OS) included all noted deaths. Disease-free survival (DFS) was defined as a noted progression of the tumour that was not detectable at the time of the resection, including locoregional recurrence and/or novel distant metastases. The treatment concepts of included patients followed internal hospital policies at each participating site, which were based on the given German guidelines at the time of diagnosis, generally meaning that all patients were intended to receive stage-adapted treatment. The definitive therapy regimen for each patient, which naturally had to be adapted to the individual clinical situation, was then decided by a multidisciplinary team of physicians during specialised tumour boards. In most cases of colon cancer this meant primary resection and adjuvant therapy in UICC Stage III. For Stage UICC II adjuvant chemotherapy was usually only administered in “high risk” patients (pT3/4, G3, <70 years, low lymph-node ratio). For rectal cancers, neoadjuvant RCTx served as the standard for advanced cases (uT3N+) of the middle or lower third of the rectum, while non-advanced cancers and tumours of the proximal third of the rectum generally received primary surgery. The local ethic committees of the Technical University of Munich (reference number: 252/16 s), the University Hospital Mainz (reference number: 837.075.16 (10394)) and Hospital Bayreuth/University Hospital Erlangen Nürnberg (reference number: 55_17 B und 239_18 Bc) approved this study [8].

### Identification of pT3 CRCs, slide selection and digitalisation

Initially, all carcinomas diagnosed as pT3 were identified from the respective cohorts and all pre-existing tumour-carrying H&E-slides were re-evaluated by the responsible pathologist of the respective site (MJ, SF, CLS) to identify the slide with the deepest level of invasion of the adipose tissue. Cases where the depth of invasion did not match with the pathology report were excluded. In the next step, the H&E slide showing the deepest invasion was digitalised. Slides were scanned in ×20 magnification using an automated whole-slide, high-throughput, brightfield slide scanner (Aperio AT2, Leica Biosystems, Wetzlar, Germany). Slides were loaded in racks with a maximum capacity of 400 slides per run. Before scanning, the scanning field (whole tissue) and focus points on the tissue were checked and adjusted manually, if necessary.

### Digital measurement of the histological depth of invasion

**Fig. 1:**
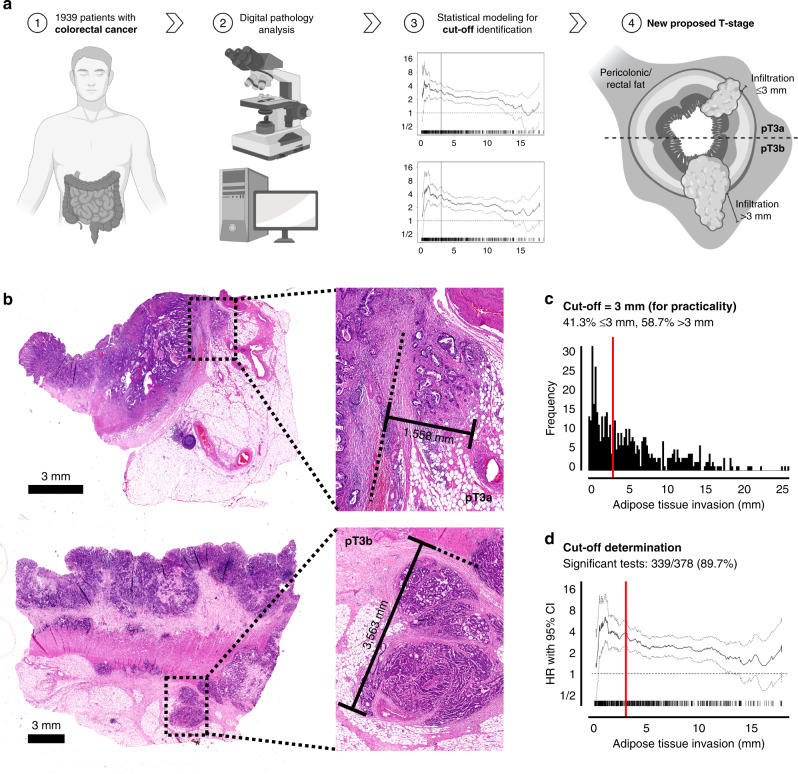
Overall workflow of the study/histological measurements/cut-off determination. **a** Graphical abstract/overview of the workflow (graphics generated using www.BioRender.com). **b** Example of the histological measurement of the histological depth of invasion depicting a pT3a case (upper panel) and a pT3b case (lower panel). The measurement was performed from the edge of the muscular layer to the deepest identifiable cancer cell within the adipose tissue. **c**, **d** Determination of the Cutoffs used for the pT3a/b subclassification guided by the biostatistical tool Cutoff finder [11]. The upper panel shows the frequency of the depth of adipose tissue invasion, the optimal cut-off is shown by the red line. The lower panel shows the hazard ratio for DSS in dependence of the cut-off point.

The digital evaluation of all CRCs was performed by an experienced GI-Pathologist (MJ; blinded to clinicopathological data at the time of the measurement) using a standardised algorithm utilising the Aperio ImageScope System (v12.4.0; Leica Biosystems, Wetzlar, Germany) [9]. For optimal orientation on the respective slides for further measurements, each case was at first evaluated in scanning magnification (0.6X) in order to localise the deepest level of invasion on the slide as well as the edge of the Tunica muscularis propria. As depicted in Fig. 1, we used the measurement system of the Aperio ImageScope System (“Ruler Tool”), to measure the exact depth of adipose tissue (in mm/µm) infiltration for each case. The measurement was performed from the last identifiable smooth muscle cell of the residual Tunica muscularis propria at the height of the focus of adipose tissue invasion until the cancer cell with the deepest localisation within the pericolic/perirectal fat. Only the deepest extent of a continuous invasion was noted, foci of tumour satellites (pN1c), foci of vascular invasion and metastatic lymph nodes in close proximity to the primary were not considered. In cases, where the muscular layer was completely destroyed by the invasive carcinoma (due to heavy desmoplastic response) or was not present on the slide of deepest invasion, the measurement was performed from the first clearly identifiable pericolonic fat cell. Some graphics used for visualisation were generated using www.BioRender.com.

### Manual measurement of the histological depth of invasion

One-hundred and twenty-five randomly selected cases from the training cohort were also manually re-measured using exactly the same measurement criteria from the digital measurements (see above). Manual measurements were performed using a clinical light microscope (Olympus BX 46) in combination with a conventional ruler by an experienced GI-pathologist (MJ), who was blinded to his initial evaluation.

### Testing for interobserver variance

Twenty randomly selected slides with different levels of tissue invasion that were digitally measured by the main observer (MJ) were given to ten different clinical pathologists with variable levels of experience ranging from residents to senior pathologists with several years of diagnostic experience. The measurements of the different pathologists were then allocated to the pT3a/b subgroups identified by the Cutoff Finder (see below) and the interobserver concordance was analysed.

Furthermore, 25 randomly selected slides that were manually re-measured by the main observer (MJ) were also given to a second clinical pathologist (MG) in order to test interobserver variability for the manual measurements.

### Statistics

Using SPSS version 28 (SPSS Institute, Chicago, IL) statistical analyses were performed using *Χ*^2^ test as well as *Χ*^2^ test for trends and Fisher’s exact test. Where necessary, the Bonferroni method was used to correct for multiple testing [10]. The interobserver variance was tested using the Spearman-correlation method. The Cutoff Finder, a publicly available biostatistical tool that represents a bundle of optimisation and visualisation methods for cut-off determination, was used to define optimal cutoffs [11]. Univariate survival analyses were performed using the Kaplan–Meier method and a log-rank test was used to assess the significance of survival differences. The Cox proportional hazard model was used for multivariate analyses. The Cohens–Kappa model was used to investigate interobserver reliability. All statistical tests were performed two-sided, and *P* values ≤0.05 were considered significant.

## Results

### Clinicopathological characteristics

The detailed cohort characteristics including all clinicopathological variables and survival data for the training cohort (Munich), validation cohort 1 (Mainz) and validation cohort 2 (Bayreuth) are given in Supplementary Tables 1–3. Briefly, the training cohort included 487 pT3 CRCs, while the validation cohorts comprised 309 (Mainz) and 240 (Bayreuth) pT3 cancers, respectively.

### Distribution of adipose tissue invasion and cut-off finding for pT3 substratification

In the training cohort (Munich, 487 pT3 CRCs) the histological depth of adipose tissue invasion ranged from 0.1 mm to 24.83 mm. In order to transform this continuous variable into dichotomous pT3 subgroups and as depicted in Fig. 1, we used the Cutoff Finder [11] to find optimal cutoffs for pT3a/b substratification. Performing statistical analyses using DSS and DFS as outcome endpoints, the Cutoff Finder calculated an optimal cut-off of 3.06 mm for the pT3a/b substratification. Worthy of note, in about 89% of the possible cutoffs that were tested for both survival endpoints, the Cutoff Finder detected statistical significance. Guided by these results, we stratified all pT3 cancers into two subgroups that were then used for further statistical analyses in all cohorts. To ensure optimal practicability, we moved the final cut-off from the proposed 3.06 mm to exactly 3 mm. Thus, pT3a CRCs were defined as cancers showing an invasion of the adipose tissue of 3 mm or less, while pT3b CRCs were defined as tumours with an infiltration depth of more than 3 mm. This pT3a/b stratification derived from the training cohort was then used for all further statistical analyses in the training cohort and the two validation cohorts.

### Interobserver variance testing

To test interobserver robustness for the digital measurements, twenty randomly selected cases from the training cohort were given to ten different clinical pathologists from different institutions, which were each blinded to clinical information and to the measurements from other pathologists. Cohens–Kappa analysis revealed an excellent interobserver agreement (*P* < 0.001; Cohens–Kappa correlation ranging from 0.9 to 0.69) among the ten different pathologists.

### Impact of the proposed pT3a/pT3b substratification on survival parameters within pT3 CRC in the training cohort and in the validation cohorts

We observed highly significant differences regarding all survival parameters between patients with pT3a and pT3b CRCs in all cohorts (DFS: *P* < 0.001 all cohorts, OS: *P* < 0.001 training cohort/validation cohort 2, *P* = 0.002 validation cohort 1, DSS: see below).

**Fig. 2:**
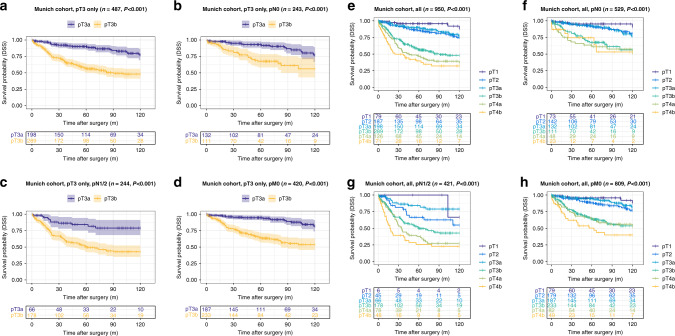
Survival analyses (log-rank test) of the proposed pT3a subclassification in the training cohort (Munich). **a**–**d** Disease-specific survival analysis of pT3a vs. pT3b CRCs within all pT3 tumours (**a**) and in pN0 (**b**), pN1/2 (**c**) and pM0 subgroups (**d**) of patients with pT3 tumours. Note the significantly worse survival for pT3b CRCs, also in the specific subgroups. **e**–**h** Disease-specific survival analysis of pT3a vs. pT3b CRCs compared to all pT stages in the overall cohort (**e**) and in pN0 (**f**), pN1/2 (**g**) and pM0 subgroups (**h**). Note that pT2 and pT3a carcinomas show an almost equal survival, while pT3b CRCs show significantly worse survival characteristics that trend towards the survival of pT4a carcinomas.

**Fig. 3:**
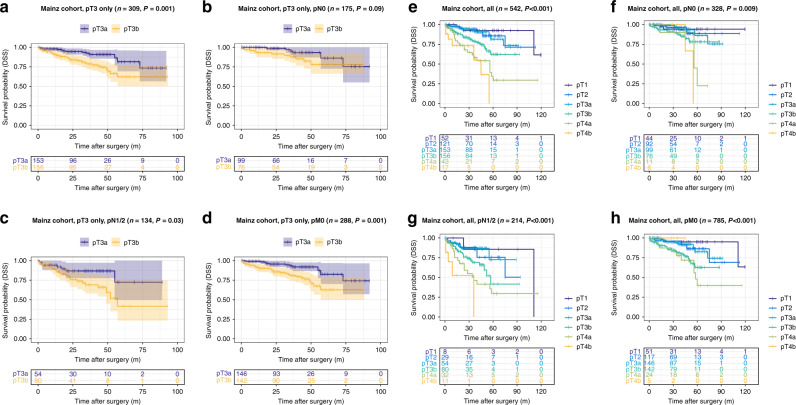
Survival analyses (log-rank test) of the proposed pT3a subclassification in validation cohort 1 (Mainz). **a**–**d** Disease-specific survival analysis of pT3a vs. pT3b CRCs within all pT3 tumours (**a**) and in pN0 (**b**), pN1/2 (**c**) and pM0 subgroups (**d**) of patients with pT3 tumours. **e**–**h** Disease-specific survival analysis of pT3a vs. pT3b CRCs compared to all pT stages in the overall cohort (**e**) and in pN0 (**f**), pN1/2 (**g**) and pM0 subgroups (**h**).

**Fig. 4:**
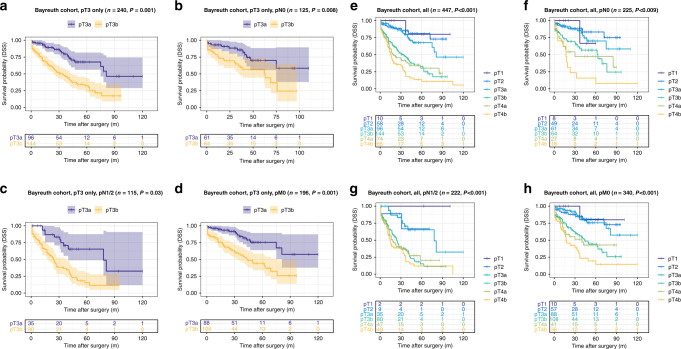
Survival analyses (log-rank test) of the proposed pT3a subclassification in validation cohort 2 (Bayreuth). **a**–**d** Disease-specific survival analysis of pT3a vs. pT3b CRCs within all pT3 tumours (**a**) and in pN0 (**b**), pN1/2 (**c**) and pM0 subgroups (**d**) of patients with pT3 tumours. **e**–**h** Disease-specific survival analysis of pT3a vs. pT3b CRCs compared to all pT stages in the overall cohort (**e**) and in pN0 (**f**), pN1/2 (**g**) and pM0 subgroups (**h**).

The pT3b group showed highly reduced disease-specific survival compared to the pT3a group (DSS training cohort (Munich): pT3a: 106.7 months vs. pT3b: 75.2 months, *P* < 0.001; validation cohort 1, pT3a: 80.6 months vs. pT3b: 69.7 months, *P* = 0.001, validation cohort 2, pT3a: 81.3 months vs. pT3b: 40.8 months, *P* < 0.001). This effect on DSS was also present in all pN subgroups (pN0 vs. pN1/2) of the training cohort (DSS pN0 subgroup, pT3a: 109.3 months vs. pT3b: 86.6 months, *P* < 0.001; pN1/2 subgroup, pT3a: 101.5 months vs. pT3b: 68.5 months, *P* < 0.001) and validation cohort 2 (validation cohort 2: pN0 subgroup, pT3a: 81.7 months vs. pT3b: 52.4 months, *P* = 0.008; pN1/2 subgroup, pT3a: 74.1 months vs. pT3b: 31.8 months, *P* < 0.001), while in validation cohort 1 only a prognostic effect for DSS in the pN1/2 subgroup was visible (validation cohort 1: pN0 subgroup, pT3a: 83.4 months vs. pT3b: 75.2 months, *P* = 0.09; pN1/2 subgroup, pT3a: 73.8 months vs. pT3b: 58.2 months, *P* = 0.03). Detailed information on the impact of the pT3a/b substratification in pT3 CRC on all survival parameters (OS, DSS, DFS) and also within other different subgroups (pM subgroups, WHO-grade subgroups) are given in Figs. 2–4 and in Supplementary Tables 4–6.

When we performed multivariate analyses of all pT3 cancers including gender, age, WHO grade, pN, pM and resection status, the pT3a/b substratification remained a highly significant and independent prognosticator in the training cohort (DSS: *P* < 0.001, HR: 2.78; Supplementary Table 7) and in both validation cohorts (DSS validation cohort 1: *P* = 0.017, HR 2.28, Supplementary Table 8/DSS validation cohort 2: *P* < 0.001, HR 2.62, Supplementary Table 9). In an additional exploratory multivariate analysis in the training cohort where also extramural vascular invasion, a strong prognostic factor in CRC [12], was included, both the pT3a/b substratification (*P* = 0.004, HR 4.93, data not shown) as well as an extramural vascular invasion (*P* = 0.014, HR 3.56, data not shown) remained independent prognostic factors.

### Impact of the “revised pT classification” including the proposed pT3a/pT3b substratification and all other pT stages on survival parameters in the training cohort and in the validation cohorts

When we investigated the prognostic relevance of our “revised pT classification” including the proposed pT3a/pT3b and all other pT Stages (1, 2, 4a/b), the proposed “revised pT classification” in general remained highly prognostic in the training cohort (Fig. 2 and details Supplementary Table 10) and in validation cohort 1 (Fig. 3 and details Supplementary Table 11) and 2 (Fig. 4 and details Supplementary Table 12), including in pN subgroups. In all cohorts, it was visible, that pT2 and pT3a CRCs shared similar survival characteristics with no significant survival differences between the two subgroups (*P* = n.s. in all cohorts for all survival parameters). In contrast, pT3b CRCs showed distinct survival characteristics compared to all other pT stages that approximated but did not exactly match those of pT4a CRCs.

This prognostic impact was again retained in multivariate analyses incorporating gender, age, WHO grade, pN, pM and resection status in the training cohort (DSS: *P* < 0.001, HR for pT3b: 5.25; Supplementary Table 13) and in both validation cohorts (DSS validation cohort 1: *P* = 0.008, HR for pT3b: 2.29; Supplementary Table 14/DSS validation cohort 2: *P* < 0.001, HR for pT3b: 6.15, Supplementary Table 15).

### Prognostic impact of the proposed pT3a/pT3b substratification and the revised pT classification including all other stages in a pooled analysis of all cohorts

**Fig. 5:**
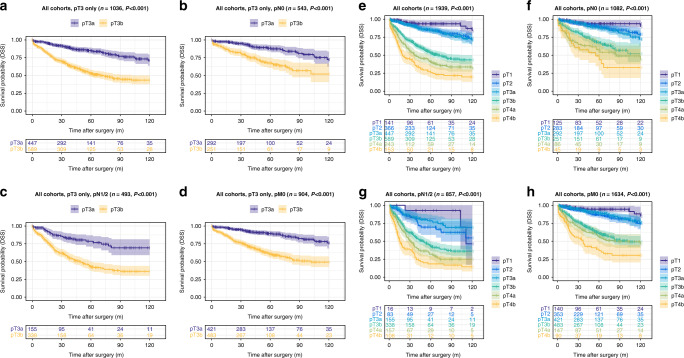
Survival analyses (log-rank test) of the proposed pT3a/b subclassification in a pooled analysis combining all CRCs from the training cohort and both validation cohorts. **a**–**d** Disease-specific survival analysis of pT3a vs. pT3b CRCs within all pT3 tumours (**a**) and in pN0 (**b**), pN1/2 (**c**) and pM0 subgroups (**d**) of patients with pT3 tumours. **e**–**h** Disease-specific survival analysis of pT3a vs. pT3b CRCs compared to all pT stages in the overall cohort (**e**) and in pN0 (**f)**, pN1/2 (**g**) and pM0 subgroups (**h**).

Finally, we performed a pooled analysis of the pT3a/b subclassification of all CRCs from the three cohorts (1939 CRCs), to even out possible inequalities between the three cohorts. We observed similar results in univariate analyses of the pooled pT3 tumours as in our single-cohort analyses (Fig. 5) and observed large survival differences between pT3a/b tumours, including in pN subgroups (DSS: *P* < 0.001).

When we performed the pooled analyses of the pT3a/b subclassification in comparison to the other pT stages, we observed similar results and again observed no survival differences between pT2 and pT3a tumours (*P* = n.s.), while pT3b tumours showed vastly reduced survival times compared to pT2 and pT3a CRCs (*P* < 0.001, Fig. 5).

Comparable results were observed in a separate analysis of the proposed pT3a/pT3b subclassification in colonic and rectal cancers (Supplementary Fig. 1). The subclassification remained highly prognostic when only pT3 tumours were analysed (colon: pT3a: 102.4 months vs. pT3b: 72.3 months; rectum: pT3a: 96.3 months vs. pT3b: 60.0 months, DSS: *P* < 0.001, respectively) and also when the pT3 subclassification was compared to the other pT stages (DSS: *P* < 0.001, respectively).

**Fig. 6:**
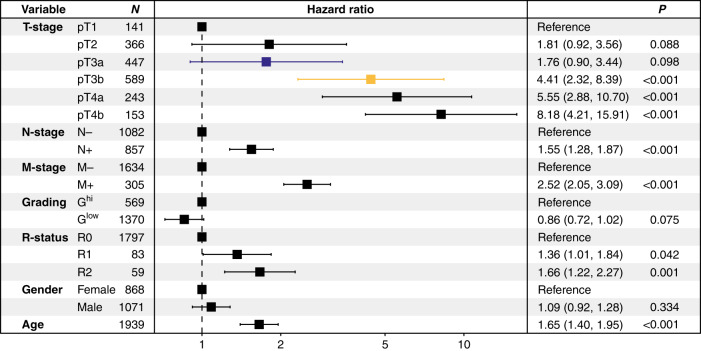
Combined multivariate analysis of the training cohort and both validation cohorts. Forest plot of the pooled multivariate analysis (Cox proportional hazard model) combining all CRCs from the training cohort and both validation cohorts including age, sex, gender, pN, pM, WHO grade, resection status and the revised pT classification.

In final multivariate analyses of the pooled cases from all cohorts, we again observed that the revised pT3 subclassification remained a highly significant predictor of survival (hazard ratio for pT3b: 4.41, *P* < 0.001), independent of pN/pM stage, WHO grade, resection status, gender or age (Fig. 6).

### Concordance of digital measurements with manual measurements using a clinical light microscope

Blinded manual re-assessment of 125 pT3 cases from the training cohort using a conventional clinical light microscope and a ruler showed an excellent concordance for the pT3a/b subcategories with the previous digital measurements. One-hundred and nineteen of the 125 cases showed a concordant allocation to the respective pT3a/b subgroups (*P* < 0.001, kappa value: 0.89, Supplementary Table 16). An exploratory analysis of interobserver variability for the manual measurements showed very good reproducibility (*P* < 0.001, kappa value: 0.82) between two clinical pathologists. In an exploratory survival analysis (log-rank test) of those 125 cases, the manual measured distinction between pT3a/b showed a similar impact on DSS as the digital measurements (*P* < 0.001, data not shown).

## Discussion

The tumour (pT), node (pN), metastasis (pM) classification (pTNM) of the Union for International Cancer Control (UICC) is the global benchmark for the staging of colorectal cancer [13]. pTNM staging translates the extent of post-operative disease into a simple formula that is understood by physicians worldwide. Therefore, it is one of the clinically most important parameters generated by pathologists, as it is the foundation of post-operative clinical decision-making and largely dictates further treatment strategies [4, 5, 13–15].

CRCs that invade the pericolic fat are categorised as pT3 without further subdivision. Therefore, the pT3 category is very broad in its current form and comprises a wide range of CRCs ranging from cancers that only superficially invade the fat to those who show an extensive infiltration of the pericolic fat with only a few µm separating them from perforating the serosa and thus, upstaging to the subsequently higher pT stage (pT4a).

In the current TNM classification [3], the concept of histological depth of invasion is already a central factor for the determination of the pT stage for oral/cutaneous/head and neck or extrahepatic bile duct cancer. Using digital pathology (and also conventional light microscopy), our analyses from three independent CRC cohorts demonstrate that further subdivision of pT3 colorectal cancer based on the histopathological depth of invasion (pT3a ≤ 3 mm; pT3b > 3 mm) generates distinct prognostic subgroups within the pT3 category. The pT3a/b subclassification retained its impact on patient survival in crucial clinicopathological subgroups (pN0; pN1/2, pM0) as well as in multivariate analyses including pN/pM, resection status, age, gender and stage. In comparison to the other pT stages, we made the interesting observation that patients with pT3a CRCs have survival characteristics that are similar to those of pT2 CRCs, with no statistical differences between these two subgroups. In contrast, we identified pT3b tumours as a distinct prognostic subgroup with highly dismal survival characteristics, that trended towards, but did not exactly match the highly unfavourable clinical course of pT4a CRCs. These findings became even clearer in the final pooled analysis of all cohorts, which we performed to even out possible inequalities between the three separate collectives.

Although there are considerable differences regarding their general clinical management [16, 17], pTNM staging of colon and rectal cancers is done using exactly the same scheme. In separate analyses of the proposed pT3a/b subclassification within the colon and rectal carcinomas, the dismal survival characteristics of pT3b tumours were equally present in both localisations.

In daily pathological practice around the world, histopathological examinations are performed using conventional light microscopes. Therefore, any classification system involving histopathology has to be feasible for light microscopy. Using the same measurement criteria used for the digital analyses, manual re-assessment of randomly selected pT3 CRCs revealed an excellent correlation between digital and conventional measurements, strongly arguing that our approach is suitable for daily pathological practice.

According to current guidelines [4–6, 18, 19], adjuvant therapy regimens are generally intended for UICC Stage III patients (pN+) or UICC Stage II patients (pT3, pT4; pN0), that show a “high risk” clinicopathological profile, which includes tumour perforation/obstruction, high WHO grade and pT4 stage. Our data indicate that the current pT3 classification does not sufficiently translate the extent of local disease spread to the clinician due to its wide range and therefore potentially withholds important prognostic information that might alter the post-operative management. Considering the dismal survival of pT3b CRCs in general and especially in the pN0 subgroup, it should be discussed whether nodal negative patients with deep invasion of the pericolic fat (“pT3b, pN0”) should also be categorised as “high risk” in upcoming guidelines [19–24].

In contrast to the binding subdivision of pT4a/b carcinomas that has been introduced with the 7th edition of the TNM classification [25, 26], the pT3 category has remained unchanged in its definition for decades. Non-binding ramifications of pT3 colonic and rectal carcinomas are mentioned in the segment “*optional proposals for testing new subcategories of TNM”* of the fourth and the recent fifth edition of the TNM supplement books [27–29], but have not made it into the daily clinicopathological routine. Our recent data are in line with the data from the few previous studies that have investigated the relevance of depth of invasion in pT3 carcinomas from smaller-sized cohorts [30–32] and represent—to our knowledge—the first large-scale multicentre approach that has investigated this topic in colonic and rectal cancer.

Our study has some limitations as it is retrospective in nature and the investigated tumours in our study have been resected and treated for a long timeframe (from 1997 to 2019). Furthermore, the exact therapy regimens are only available for a subset of patients and are therefore not included in our analyses. However, as CRC patients generally receive stage-adapted treatment, we believe that the main therapeutic groups are adequately mirrored within our various subgroup analyses. Finally, additional studies have to validate our proposed Cutoff-point (3 mm), that has been biostatistically generated using the data from our training cohort, although it has already been validated in two independent validation cohorts.

In conclusion, our data clearly show that the extent of adipose tissue infiltration is of major prognostic significance for CRC patients and therefore, implementation in routine histopathology reports for CRC resection specimens should be considered. In order to incorporate this finding into the clinically important pTNM classification and to optimise post-operative clinical decision-making for patients with pT3 CRCs, we propose a subdivision into pT3a/b CRCs based on a Cutoff-point of 3 mm.

## Supplementary information

Supplementary material

aj-checklist

## Data Availability

All data relevant to this study are given in the main paper including figures, tables and Supplemental Files. The tissue investigated for this study is archived in the Institute of Pathology of the Technical University of Munich, the Institute of Pathology of the University Hospital Mainz and the Institute of Pathology of the Hospital Bayreuth.
