# Supplementary material for: pT3 colorectal cancer revisited: a multicentric study on the histological depth of invasion in more than 1000 pT3 carcinomas—proposal for a new pT3a/pT3b subclassification
Source: Br J Cancer. 2022 Jul 21;127(7):1270–8. doi: 10.1038/s41416-022-01889-1 (PMC9519960; doi:10.1038/s41416-022-01889-1)
Supplement: Supplementary file 1 — Supplementary material [file 41416_2022_1889_MOESM1_ESM.pdf]

Supplementary Figure 1

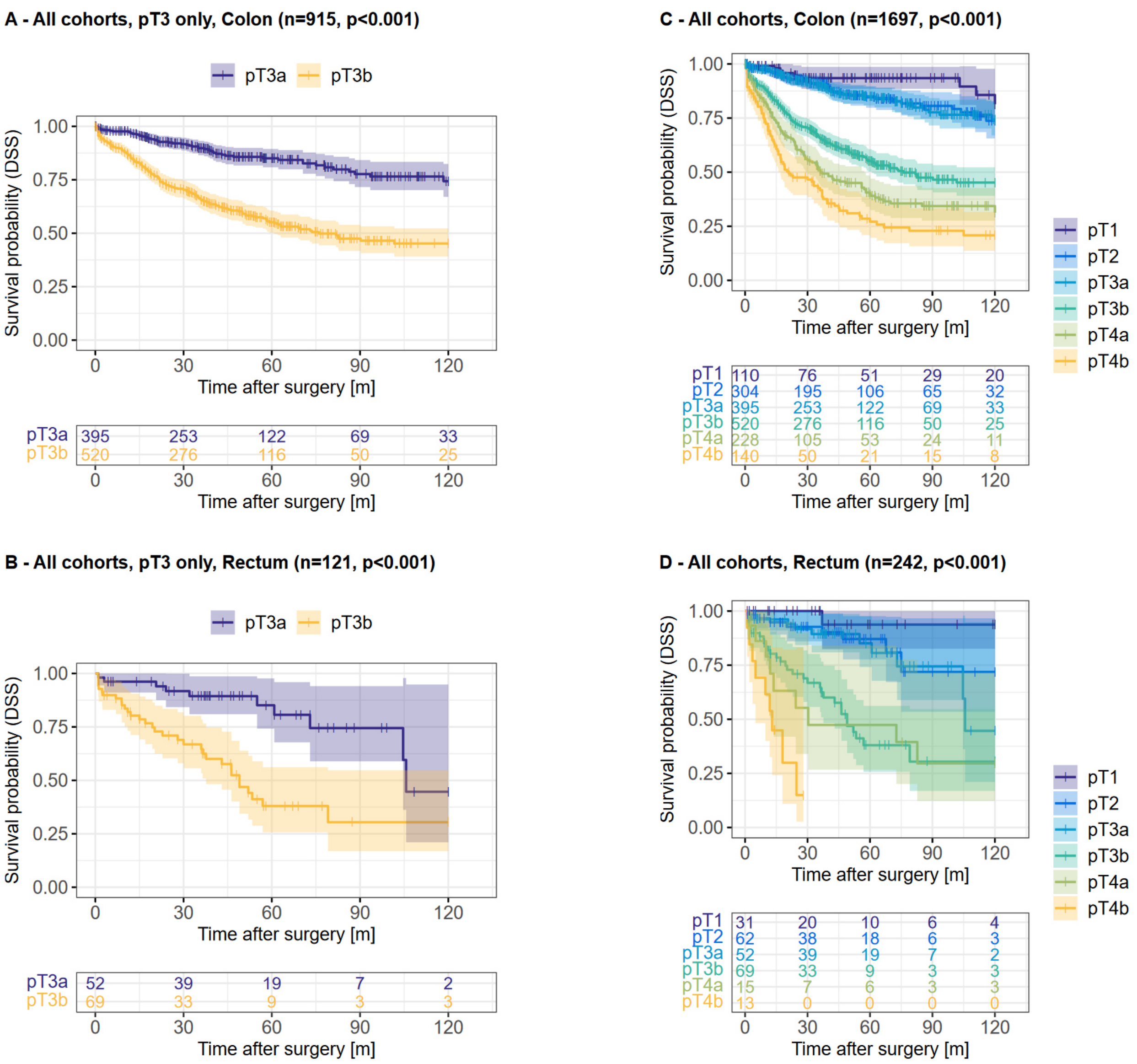

Supplementary Table 1		Overall n (%)	Mean overall survival (SE) [months]	*p-value*	Mean disease specific survival (SE) [months]	*p-value*	Mean disease free survival (SE) [months]	*p-value*
**Age**				***<0.001***		***0.04***		***0.76***
	below median	*471 (49.5%)*	85.87 (2.2)		90.60 (2.2)		80.89 (2.4)	
	above median	*479 (50.5%)*	72.29 (2.3)		84.06 (2.4)		83.50 (2.5)	
**Sex**				***0.32***		***0.69***		***0.44***
	male	*543 (57.2%)*	77.79 (2.1)		88.04 (2.1)		82.85 (2.3)	
	female	*407 (42.8%)*	81.05 (2.5)		86.58 (2.5)		80.95 (2.8)	
**pT**				***<0.001***		***<0.001***		***<0.001***
	1	*79 (8.3%)*	97.68 (4.8)		115.54 (2.9)		111.58 (3.7)	
	2	*187 (19.7%)*	93.33 (3.2)		103.92 (2.8)		99.92 (3.1)	
	3	*487 (51.3%)*	80.13 (2.2)		88.23 (2.2)		81.04 (2.4)	
	4a	*126 (13.3%)*	61.58 (4.5)		63.72 (4.6)		53.70 (4.8)	
	4b	*71 (7.5%)*	46.66 (5.8)		53.65 (6.4)		53.93 (7.0)	
**pN**				***<0.001***		***<0.001***		***<0.001***
	0	*529 (55.7%)*	89.76 (2.0)		101.78 (1.8)		100.42 (2.0)	
	1	*264 (27.8%)*	75.59 (3.1)		80.50 (3.1)		71.97 (3.4)	
	2	*157 (16.5%)*	49.57 (4.0)		52.45 (4.1)		40.55 (4.0)	
**pM**				***<0.001***		***<0.001***		***<0.001***
	0	*809 (85.2%)*	86.69 (1.7)		96.38 (1.6)		91.63 (1.8)	
	1	141 (14.8%)	39.46 (3.5)		41.42 (3.7)		33.63 (3.6)	
**UICC Stage**				***<0.001***		***<0.001***		***<0.001***
	1	*212 (22.3%)*	97.09 (2.9)		111.65 (2.0)		110.02 (2.4)	
	2	*300 (31.6%)*	86.61 (2.8)		97.15 (2.6)		94.74 (2.8)	
	3	*293 (30.8%)*	80.01 (2.9)		85.82 (2.9)		74.24 (3.2)	
	4	*145 (15.3%)*	38.71 (3.4)		40.65 (3.6)		32.79 (3.6)	
**WHO grade**				***<0.001***		***<0.001***		***<0.001***
	low-grade	*645 (67.9%)*	86.11 (1.9)		94.84 (1.8)		90.06 (2.0)	
	high-grade	*305 (32.1%)*	65.01 (2.9)		72.22 (3.0)		66.53 (3.2)	
**Resection margin**				***<0.001***		***<0.001***		***<0.001***
	R0	*883 (92.9%)*	82.83 (1.6)		91.83 (1.6)		87.07 (1.8)	
	R1	*40 (4.2%)*	41.82 (7.5)		43.37 (7.8)		31.44 (6.9)	
	R2	*27 (2.8%)*	22.13 (3.3)		22.13 (3.3)		21.87 (4.0)	
**Tumour localisation**				***0.55***		***0.61***		***0.83***
	Coecum	*137 (14.4%)*	76.59 (4.3)		86.69 (4.3)		83.61 (4.5)	
	Ascendens	*240 (25.3%)*	79.00 (3.3)		88.04 (3.3)		80.01 (3.5)	
	Transversum	*75 (7.9%)*	74.14 (6.1)		85.09 (6.1)		84.33 (6.3)	
	Descendens	*89 (9.4%)*	74.75 (4.3)		80.65 (5.3)		78.09 (5.6)	
	Sigmoid	*305 (32.1%)*	83.89 (2.8)		90.73 (2.8)		84.00 (3.0)	
	Rectum	*104 (10.9%)*	76.97 (4.8)		85.14 (4.8)		78.05 (5.4)	

Supplementary Table 2	Overall n (%)	Mean overall survival (SE) [months]	*p-value*	Mean disease specific survival (SE) [months]	*p-value*	Mean disease free survival (SE) [months]	*p-value*
**Age**			***0.001***		**0.01**		***0.10***
below median	*263 (48.5%)*	100.51 (5.2)		102.82 (5.1)		84.91 (4.5)	
above median	*279 (51.2%)*	80.05 (4.1)		84.46 (3.8)		75.07 (4.5)	
**Sex**			***0.31***		**0.68**		***0.43***
male	*289 (53.3%)*	88.84 (5.4)		94.47 (5.5)		77.71 (4.4)	
female	*253 (46.7%)*	89.01 (4.0)		89.72 (4.0)		79.51 (5.8)	
**pT**			***<0.001***		**<0.001**		***<0.001***
1	*52 (9.6%)*	107.79 (9.1)		117.04 (6.9)		107.45 (5.1)	
2	*121 (22.3%)*	90.02 (6.1)		95.91 (5.9)		86.62 (6.8)	
3	*309 (57.0%)*	74.41 (2.5)		74.92 (2.5)		73.98 (6.2)	
4a	*43 (7.9%)*	58.25 (7.9)		59.91 (8.0)		50.76 (7.7)	
4b	*17 (3.1%)*	32.67 (6.2)		34.67 (6.3)		16.15 (3.4)	
**pN**			***<0.001***		**<0.001**		***<0.001***
0	*328 (60.5%)*	103.73 (4.7)		109.66 (4.4)		96.62 (3.6)	
1	*136 (25.1%)*	87.27 (5.4)		87.95 (5.4)		74.31 (5.8)	
2	*78 (14.4%)*	38.56 (4.7)		39.18 (4.7)		29.56 (3.5)	
**pM**			***<0.001***		**<0.001**		***<0.001***
0	*485 (89.5%)*	97.41 (4.4)		101.42 (4.4)		85.17 (3.7)	
1	*57 (10.5%)*	39.64 (5.6)		40.68 (5.7)		24.96 (3.3)	
**UICC Stage**			***<0.001***		**<0.001**		***<0.001***
1	*135 (24.9%)*	107.66 (7.6)		122.63 (3.6)		109.74 (3.4)	
2	*182 (33.6%)*	77.69 (2.9)		78.54 (2.9)		74.46 (3.0)	
3	*168 (31.0%)*	79.75 (5.2)		80.26 (5.2)		66.66 (5.1)	
4	*57 (10.5%)*	39.64 (5.6)		40.68 (5.7)		24.96 (3.3)	
**WHO grade**			***0.02***		**0.002**		***0.10***
low-grade	*388 (71.6%)*	100.54 (4.3)		106.03 (4.1)		81.38 (4.7)	
high-grade	*154 (28.4%)*	77.31 (5.2)		77.31 (5.2)		73.09 (5.2)	
**Resection margin**			***0.01***		**0.001**		***<0.001***
R0	*523 (96.5%)*	93.28 (4.5)		97.05 (4.5)		79.83 (3.7)	
R1	*17 (3.1%)*	61.63 (14.2)		61.63 (14.2)		52.86 (14.4)	
R2	*2 (0.4)*	20.00 (3.5)		20.00 (3.5)		19.50 (4.5)	
**Tumour localisation**			***0.20***		**0.33**		***0.28***
Coecum	*75 (13.8%)*	59.05 (3.7)		62.68 (3.4)		53.38 (3.4)	
Ascendens	*148 (27.3%)*	87.70 (5.0)		93.21 (4.0)		83.65 (4.4)	
Transversum	*48 (8.9%)*	83.27 (8.6)		83.27 (8.6)		71.15 (8.1)	
Descendens	*74 (13.7%)*	108.88 (6.6)		108.88 (6.6)		86.61 (7.5)	
Sigma	*92 (17.0%)*	61.01 (4.9)		63.97 (4.9)		55.53 (4.4)	
Rectum	*105 (19.4%)*	86.65 (7.7)		87.55 (7.8)		80.77 (7.4)	

Supplementary Table 3	Overall n (%)	Mean overall survival (SE) [months]	*p-value*	Mean disease specific survival (SE) [months]	*p-value*	Mean disease free survival (SE) [months]	*p-value*
**Age**			***0.001***		***0.33***		***0.51***
below median	*221 (49.4%)*	49.05 (3.2)		57.71 (3.9)		53.35 (3.8)	
above median	*226 (50.6%)*	34.92 (2.4)		49.30 (3.4)		47.02 (3.3)	
**Sex**			***0.62***		***0.71***		***0.48***
male	*239 (53.5%)*	41.19 (2.8)		54.55 (3.9)		49.49 (3.6)	
female	*208 (46.5%)*	42.22 (2.7)		52.28 (3.3)		50.63 (3.4)	
**pT**			***<0.001***		***<0.001***		***<0.001***
1	*10 (2.2%)*	47.12 (13.7)		88.20 (11.4)		88.20 (11.4)	
2	*58 (13.0%)*	56.89 (5.0)		79.39 (5.0)		75.93 (5.3)	
3	*240 (53.7%)*	45.65 (3.0)		58.22 (4.0)		55.77 (4.0)	
4a	*74 (16.6%)*	35.72 (4.5)		38.25 (4.7)		33.42 (4.2)	
4b	*65 (14.5%)*	24.46 (3.9)		28.06 (4.6)		28.93 (4.5)	
**pN**			***0.001***		***<0.001***		***<0.001***
0	*225 (50.3%)*	48.45 (3.0)		72.41 (4.3)		68.81 (4.2)	
1	*113 (25.3%)*	38.34 (3.5)		44.70 (4.1)		41.63 (4.0)	
2	*109 (24.4%)*	32.86 (4.0)		35.63 (4.5)		31.08 (4.1)	
**pM**			***<0.001***		***<0.001***		***<0.001***
0	*340 (76.1%)*	48.61 (2.5)		69.75 (3.5)		67.24 (3.5)	
1	*107 (23.9%)*	23.12 (2.6)		24.13 (2.7)		21.09 (2.3)	
**UICC Stage**			***<0.001***		***<0.001***		***<0.001***
1	*54 (12.1%)*	55.16 (5.2)		80.00 (5.1)		78.12 (5.2)	
2	*156 (34.9%)*	49.41 (3.9)		69.68 (5.3)		66.72 (5.2)	
3	*130 (29.1%)*	46.77 (4.4)		59.78 (5.4)		57.53 (5.5)	
4	*107 (23.9%)*	23.00 (2.5)		23.99 (2.6)		20.96 (2.2)	
**WHO grade**			***0.02***		***0.03***		***0.02***
low-grade	*337 (75.4%)*	44.24 (2.4)		58.45 (3.4)		54.78 (3.2)	
high-grade	*110 (24.6%)*	33.92 (3.4)		41.68 (4.0)		38.94 (3.9)	
**Resection margin**			***<0.001***		***<0.001***		***<0.001***
R0	*391 (87.5%)*	45.29 (2.3)		61.97 (3.3)		58.93 (3.3)	
R1	*26 (5.8%)*	17.56 (3.9)		17.56 (3.9)		16.50 (3.5)	
R2	*30 (6.7%)*	24.47 (4.4)		25.40 (4.4)		25.63 (4.3)	
**Tumour localisation**			***0.02***		***0.18***		***0.04***
Coecum	*81 (18.1%)*	40.98 (5.2)		57.24 (6.2)		52.52 (6.2)	
Ascendens	*135 (30.2%)*	49.74 (4.2)		75.64 (4.7)		61.00 (5.2)	
Transversum	*81 (18.1%)*	36.92 (3.9)		65.89 (7.0)		46.41 (5.0)	
Descendens	*54 (12.1%)*	41.58 (5.8)		59.98 (9.8)		42.81 (6.7)	
Sigmoid	*63 (14.1%)*	43.62 (5.7))		61.88 (7.7)		50.42 (6.7)	
Rectum	*33 (7.4%)*	26.19 (4.8)		34.65 (9.3)		32.63 (6.5)	

**Supplementary Table 4**		***Overall n (%)***	**Mean overall survival (SE) [months]**	*p-value*	**Mean disease specific survival (SE) [months]**	*p-value*	**Mean disease free survival (SE) [months]**	*p-value*
All pT3 Tumors				***<0.001***		***<0.001***		***<0.001***
	pT3a	198 (40.7%)	94.73 (3.0)		106.70 (2.5)		100.08 (3.1)	
	pT3b	289 (59.3%)	69.66 (3.0)		75.22 (3.1)		67.39 (3.3)	
WHO grade: low-grade subcohort				**0.003**		***<0.001***		***<0.001***
	pT3a	142 (43.4%)	95.49 (3.5)		108.19 (2.8)		101.32 (3.5)	
	pT3b	185 (56.6%)	78.20 (3.7)		83.99 (3.7)		75.59 (4.1)	
WHO grade: high-grade subcohort				**<0.001**		***<0.001***		***<0.001***
	pT3a	56 (35.0%)	92.60 (5.8)		102.89 (5.0)		97.02 (6.1)	
	pT3b	104 (65.0%)	54.46 (4.9)		59.39 (5.2)		52.88 (5.3)	
pN0 subcohort				***<0.001***		***<0.001***		***0.008***
	pT3a	132 (54.3%)	97.79 (3.4)		109.27 (2.7)		105.13 (3.3)	
	pT3b	111 (45.7%)	76.07 (4.8)		86.60 (4.9)		88.97 (5.0)	
pN1/2 subcohort				***0.003***		***<0.001***		***<0.001***
	pT3a	66 (27.0%)	88.82 (5.8)		101.47 (5.1)		89.92 (6.2)	
	pT3b	178 (73.0%)	65.40 (3.9)		68.53 (4.0)		55.51 (4.0)	
pM0				***<0.001***		***<0.001***		***<0.001***
	pT3a	187 (44.5%)	98.19 (2.9)		111.20 (2.1)		104.36 (2.9)	
	pT3b	233 (55.5%)	75.08 (3.4)		82.15 (3.4)		74.76 (3.7)	
pM1				***0.827***		***0.827***		***0.956***
	pT3a	11 (16.4%)	37.04 (7.6)		37.04 (7.6)		29.67 (8.2)	
	pT3b	56 (83.6%)	48.74 (6.3)		49.58 (6.4)		39.36 (6.3)	

Supplementary Table 5		***Overall n (%)***	**Mean overall survival (SE) [months]**	*p-value*	**Mean disease specific survival (SE) [months]**	*p-value*	**Mean disease free survival (SE) [months]**	*p-value*
All pT3 Tumors				***0.002***		***0.001***		***<0.001***
	pT3a	153 (49.5%)	80.09 (3.2)		80.61 (3.2)		87.09 (8.0)	
	pT3b	156 (50.5%)	69.17 (3.5)		69.67 (3.5)		55.08 (3.3)	
WHO grade: low-grade subcohort				**0.008**		***0.007***		***<0.001***
	pT3a	111 (50.9%)	81.05 (3.4)		81.79 (3.4)		87.79 (8.3)	
	pT3b	107 (49.1%)	71.06 (4.1)		71.88 (4.1)		54.79 (4.0)	
WHO grade: high-grade subcohort				**0.177**		***0.177***		***0.045***
	pT3a	42 (46.2%)	68.12 (6.8)		68.12 (6.8)		66.28 (6.8)	
	pT3b	49 (53.8%)	57.61 (4.0)		57.61 (4.0)		51.31 (4.1)	
pN0 subcohort				***0.096***		***0.089***		***0.034***
	pT3a	99 (56.6%)	82.56 (3.5)		83.40 (3.5)		79.84 (3.7)	
	pT3b	76 (43.4%)	74.20 (3.6)		75.20 (3.5)		68.44 (3.9)	
pN1/2 subcohort				***0.031***		***0.031***		***<0.001***
	pT3a	54 (40.3%)	73.82 (5.8)		73.82 (5.8)		82.23 (10.0)	
	pT3b	80 (59.7%)	58.24 (5.9)		58.24 (5.9)		34.79 (2.6)	
pM0				***0.002***		***0.001***		***<0.001***
	pT3a	146 (50.7%)	81.03 (3.2)		81.59 (3.1)		89.31 (8.1)	
	pT3b	142 (49.3%)	67.00 (3.4)		67.50 (3.4)		56.62 (3.4)	
pM1				***0.879***		***0.879***		***0.388***
	pT3a	7 (33.3%)	24.86 (5.5)		24.86 (5.5)		15.86 (4.5)	
	pT3b	14 (66.7%)	56.06 (12.1)		56.06 (12.1)		33.11 (7.7)	

Supplementary Table 6		*Overall n (%)*	Mean overall survival (SE) [months]	*p-value*	Mean disease specific survival (SE) [months]	*p-value*	Mean disease free survival (SE) [months]	*p-value*
All pT3 Tumors				***0.001***		***<0.001***		***<0.001***
	pT3a	96 (40.0%)	57.45 (5.2)		81.27 (6.5)		77.94 (6.5)	
	pT3b	144 (60.0%)	36.23 (3.0)		40.79 (3.4)		37.90 (3.5)	
WHO grade: low-grade subcohort				**0.001**		***<0.001***		***<0.001***
	pT3a	82 (42.1%)	58.95 (5.6)		81.40 (6.8)		78.41 (6.8)	
	pT3b	113 (57.9%)	36.22 (3.4)		41.40 (4.0)		38.00 (4.0)	
WHO grade: high-grade subcohort				**0.255**		***0.032***		***0.032***
	pT3a	14 (31.1%)	39.10 (4.6)		45.55 (3.8)		42.63 (4.3)	
	pT3b	31 (68.9%)	34.65 (5.7)		39.13 (6.4)		35.96 (6.6)	
pN0 subcohort				***0.393***		***0.008***		***0.034***
	pT3a	61 (48.8%)	53.65 (6.1)		81.68 (7.0)		77.42 (7.0)	
	pT3b	64 (51.2%)	44.74 (4.7)		52.43 (5.6)		53.0 (6.0)	
pN1/2 subcohort				***<0.001***		***<0.001***		***<0.001***
	pT3a	35 (30.4%)	64.71 (9.2)		74.09 (9.7)		70.33 (10.3)	
	pT3b	80 (69.6%)	29.96 (3.5)		31.75 (3.7)		26.04 (3.4)	
pM0				***0.012***		***<0.001***		***<0.001***
	pT3a	88 (44.9%)	61.35 (5.7)		90.31 (6.8)		86.56 (6.7)	
	pT3b	108 (55.1%)	42.08 (3.7)		49.23 (4.4)		49.18 (4.6)	
pM1				***0.418***		***0.418***		***0.175***
	pT3a	8 (18.2%)	28.38 (8.6)		28.38 (8.6)		26.88 (8.4)	
	pT3b	36 (81.8%)	21.72 (3.3)		21.72 (3.3)		16.56 (2.7)	

Supplementary Table 7		HR (DSS)	lower CI (95%)	upper CI (95%)	*p-value*
**revised pT3**					**<0.001**
	pT3a	1.00			
	pT3b	2.78	1.76	4.40	
**Gender**					***0.119***
	male	1.00			
	female	0.75	0.52	1.10	
**WHO-grade**					**0.016**
	Low grade	1.00			
	High grade	1.54	1.10	2.18	
**pN**					**0.104**
	N0	1.00			
	N1	1.07	0.70	1.64	
	N2	1.51	0.92	2.48	
**pM**					***<0.001***
	M0	1.00			
	M1	2.42	1.57	3.71	
**Resection margin**					**0.137**
	R0	1.00			
	R1	1.30	0.58	2.91	
	R2	1.78	0.83	3.82	
**Age group**					**0.074**
	Below median	1.00			
	Median and above	1.38	0.97	1.95	

Supplementary Table 8	HR (DSS)	lower CI (95%)	upper CI (95%)	*p-value*
**revised pT3**				**0.017**
pT3a	1.00			
pT3b	2.28	1.16	4.48	
**Gender**				***0.331***
male	1.00			
female	0.73	0.39	1.37	
**WHO-grade**				**0.859**
Low grade	1.00			
High grade	0.95	0.51	1.76	
**pN**				**<0.001**
N0	1.00			
N1	1.40	0.64	3.09	
N2	3.90	1.93	7.89	
**pM**				***0.036***
M0	1.00			
M1	2.75	1.07	7.11	
**Resection margin**				**0.195**
R0	1.00			
R1	0.85	0.19	3.76	
R2	4.62	0.46	46.78	
**Age group**				**0.001**
Below median	1.00			
Median and above	3.46	1.69	7.07	

Supplementary Table 9	HR (DSS)	lower CI (95%)	upper CI (95%)	*p-value*
**revised pT3**				**<0.001**
T3a	1.00			
T3b	2.62	1.60	4.28	
**Gender**				***0.779***
female	1.00			
male	1.06	0.71	1.59	
**WHO-grade**				**0.786**
Low grade	1.00			
High grade	0.93	0.56	1.55	
**pN**				**0.151**
N1	1.00			
N2	1.13	0.66	1.94	
N3	1.58	0.85	2.94	
**pM**				***0.002***
M0	1.00			
M1	2.73	1.44	5.20	
**Resection margin**				**0.364**
R0	1.00			
R1	1.51	0.62	3.69	
R2	1.20	0.53	2.71	
**Age group**				**0.002**
Below median	1.00			
Median and above	1.93	1.27	2.91	

Supplementary Table 10		*Overall n (%)*	**Mean overall survival (SE) [months]**	*p-value*	**Mean disease specific survival (SE) [months]**	*p-value*	**Mean disease free survival (SE) [months]**	*p-value*
revised pT classification				***<0.001***		***<0.001***		***<0.001***
	pT1	79 (8.3%)	97.68 (4.8)		115.54 (2.9)		111.58 (3.7)	
	pT2	187 (19.7%)	93.33 (3.2)		103.92 (2.8)		99.92 (3.1)	
	pT3a	198 (20.9%)	94.73 (3.0)		106.70 (2.5)		100.08 (3.1)	
	pT3b	289 (30.4%)	69.66 (3.0)		75.22 (3.1)		67.39 (3.3)	
	pT4a	126 (13.3%)	61.58 (4.5)		63.72 (4.6)		53.70 (4.8)	
	pT4b	71 (7.5%)	46.66 (5.8)		53.65 (6.4)		53.93 (7.0)	
WHO grade: low-grade subcohort				**<0.001**		***<0.001***		***<0.001***
	pT1	66 (10.2%)	100.80 (5.1)		116.48 (3.0)		113.07 (3.8)	
	pT2	148 (22.9%)	95.31 (3.6)		105.23 (3.0)		99.90 (3.5)	
	pT3a	142 (22.0%)	95.49 (3.5)		108.19 (2.8)		101.32 (3.5)	
	pT3b	185 (28.7%)	78.20 (3.7)		83.99 (3.7)		75.59 (4.1)	
	pT4a	68 (10.5%)	72.34 (6.2)		74.43 (6.3)		63.46 (6.6)	
	pT4b	36 (5.6%)	49.12 (7.9)		57.30 (9.0)		63.28 (10.2)	
WHO grade: high-grade subcohort				**<0.001**		***<0.001***		***<0.001***
	pT1	13 (4.3%)	82.77 (13.1)		110.63 (8.9)		102.34 (11.3)	
	pT2	39 (12.8%)	86.50 (7.3)		99.54 (6.4)		99.79 (6.9)	
	pT3a	56 (18.4%)	92.60 (5.8)		102.89 (5.0)		97.02 (6.1)	
	pT3b	104 (34.1%)	54.46 (4.9)		59.39 (5.2)		52.88 (5.3)	
	pT4a	58 (19.0%)	48.61 (6.0)		50.79 (6.3)		42.63 (6.5)	
	pT4b	35 (11.5%)	43.70 (8.4)		49.65 (9.2)		44.22 (9.4)	
pN0 subcohort				***<0.001***		***<0.001***		***<0.001***
	pT1	73 (13.8%)	97.16 (5.1)		115.18 (3.1)		112.24 (3.8)	
	pT2	142 (26.8%)	96.76 (3.5)		109.37 (2.6)		106.64 (3.0)	
	pT3a	132 (25.0%)	97.79 (3.4)		109.27 (2.7)		105.13 (3.3)	
	pT3b	111 (21.0%)	76.07 (4.8)		86.60 (4.9)		88.97 (5.0)	
	pT4a	48 (9.1%)	75.03 (7.6)		79.43 (7.7)		71.87 (8.0)	
	pT4b	23 (4.3%)	62.07 (10.3)		80.58 (11.6)		73.88 (12.4)	
pN1/2 subcohort				***<0.001***		***<0.001***		***<0.001***
	pT1	6 (1.4%)	101.61 (16.8)		114.37 (4.6)		101.76 (16.3)	
	pT2	45 (10.7%)	82.17 (7.6)		86.03 (7.4)		78.07 (8.1)	
	pT3a	66 (15.7%	88.82 (5.8)		101.47 (5.1)		89.92 (6.2)	
	pT3b	178 (42.3%)	65.40 (3.9)		68.53 (4.0)		55.51 (4.0)	
	pT4a	78 (18.5%)	52.84 (5.3)		53.66 (5.4)		42.85 (5.6)	
	pT4b	48 (11.4%)	39.16 (6.6)		42.05 (7.1)		44.89 (8.1)	
pM0				***<0.001***		***<0.001***		***<0.001***
	pT1	79 (9.8%)	97.68 (4.8)		115.54 (2.9)		111.58 (3.7)	
	pT2	179 (22.1%)	95.18 (3.2)		106.40 (2.6)		102.26 (3.1)	
	pT3a	187 (23.1%)	98.19 (2.9)		111.20 (2.1)		104.36 (2.9)	
	pT3b	233 (28.8%)	75.08 (3.4)		82.15 (3.4)		74.78 (3.7)	
	pT4a	82 (10.1%)	78.05 (5.6)		80.73 (5.6)		71.04 (6.1)	
	pT4b	49 (6.1%)	58.75 (7.4)		64.63 (7.9)		62.03 (8.3)	
pM1				***0.029***		***0.158***		***0.284***
	pT1	0 (0.0%)	-		-		-	
	pT2	8 (5.7%)	49.08 (15.0)		49.08 (15.0)		47.28 (16.0)	
	pT3a	11 (7.8%)	37.04 (7.6)		37.04 (7.6)		29.67 (8.2)	
	pT3b	56 (39.7%)	48.74 (6.3)		49.58 (6.4)		39.36 (6.3)	
	pT4a	44 (31.2%)	29.74 (3.7)		30.34 (3.8)		20.75 (3.2)	
	pT4b	22 (15.6%)	21.19 (5.2)		26.94 (7.3)		28.85 (8.6)	

Supplementary Table 11		Overall n (%)	Mean overall survival (SE) [months]	p-value	Mean disease specific survival (SE) [months]	p-value	Mean disease free survival (SE) [months]	p-value
revised pT classification				***<0.001***		***<0.001***		***<0.001***
	pT1	52 (9.6%)	107.79 (9.1)		117.04 (6.9)		107.45 (5.1)	
	pT2	121 (22.3%)	90.02 (6.1)		95.91 (5.9)		86.62 (6.8)	
	pT3a	153 (28.2%)	80.09 (3.2)		80.61 (3.2)		87.09 (8.0)	
	pT3b	156 (28.8%)	69.17 (3.5)		69.67 (3.5)		55.08 (3.3)	
	pT4a	43 (7.9%)	55.87 (7.6)		57.37 (7.7)		47.91 (7.8)	
	pT4b	17 (3.1%)	34.88 (6.7)		37.65 (6.7)		30.42 (7.0)	
WHO grade: low-grade subcohort				**<0.001**		***<0.001***		***<0.001***
	pT1	44 (11.3%)	105.03 (9.1)		116.97 (3.0)		114.03 (4.1)	
	pT2	95 (24.5%)	87.22 (6.6)		94.21 (6.4)		82.41 (7.4)	
	pT3a	111 (28.6%)	81.05 (3.4)		81.79 (3.4)		87.79 (8.3)	
	pT3b	107 (27.6%)	71.06 (4.1)		71.88 (4.1)		54.79 (4.0)	
	pT4a	22 (5.7%)	54.94 (8.5)		57.88 (8.6)		47.02 (8.6)	
	pT4b	9 (2.3%)	32.00 (6.1)		35.87 (5.7)		21.31 (5.1)	
WHO grade: high-grade subcohort				**<0.001**		***<0.001***		***<0.001***
	pT1	8 (5.2%)	89.46 (15.7)		89.46 (15.7)		87.46 (16.87)	
	pT2	26 (16.9%)	63.38 (3.1)		63.38 (3.1)		63.24 (3.2)	
	pT3a	42 (27.3%)	68.12 (6.8)		68.12 (6.8)		66.28 (6.8)	
	pT3b	49 (31.8%)	57.61 (4.0)		57.61 (4.0)		51.31 (4.1)	
	pT4a	21 (13.6%)	48.17 (8.7)		48.17 (8.7)		40.66 (9.9)	
	pT4b	8 (5.2%)	34.25 (8.5)		34.25 (8.5)		35.05 (10.0)	
pN0 subcohort				***0.085***		***0.009***		***<0.001***
	pT1	44 (13.4%)	102.37 (9.2)		113.96 (4.2)		111.30 (4.8)	
	pT2	92 (28.0%)	93.55 (6.9)		104.29 (3.9)		102.68 (4.2)	
	pT3a	99 (30.2%)	82.55 (3.5)		83.40 (3.5)		79.84 (3.7)	
	pT3b	76 (23.2%)	74.20 (3.6)		75.20 (3.5)		68.44 (3.9)	
	pT4a	11 (3.4%)	56.43 (5.6)		56.43 (5.6)		45.32 (8.9)	
	pT4b	6 (1.8%)	51.67 (3.8)		51.67 (3.8)		47.33 (8.6)	
pN1/2 subcohort				***<0.001***		***<0.001***		***<0.001***
	pT1	8 (3.7%)	98.43 (16.5)		98.43 (16.5)		98.43 (16.5)	
	pT2	29 (13.6%)	72.47 (7.2)		72.47 (7.2)		57.81 (6.4)	
	pT3a	54 (25.2%)	73.82 (5.8)		73.82 (5.8)		82.23 (10.0)	
	pT3b	80 (37.4%)	58.24 (5.9)		58.24 (5.9)		34.79 (2.6)	
	pT4a	32 (15.0%)	50.61 (8.8)		52.64 (9.0)		43.60 (8.68)	
	pT4b	11 (5.1%)	18.33 (6.0)		21.16 (6.4)		18.53 (6.0)	
pM0				***0.002***		***<0.001***		***<0.001***
	pT1	51 (10.5%)	110.07 (9.0)		112.00 (4.2)		109.73 (4.7)	
	pT2	117 (24.1%)	89.30 (6.7)		95.39 (6.7)		88.06 (6.9)	
	pT3a	146 (30.1%)	81.03 (3.2)		81.59 (3.1)		89.31 (8.1)	
	pT3b	142 (29.3%)	67.00 (3.4)		67.50 (3.4)		56.62 (3.4)	
	pT4a	24 (4.9%)	71.03 (9.7)		71.07 (9.7)		69.79 (10.8)	
	pT4b	5 (1.0%)	37.00 (7.2)		45.00 (0.0)		21.87 (6.9)	
pM1				***0.326***		***0.372***		***0.592***
	pT1	1 (1.8%)	23.00 (0.0)		23.00 (0.0)		23.00 (0.0)	
	pT2	4 (7.0%)	56.33 (15.2)		56.33 (15.24)		21.38 (3.7)	
	pT3a	7 (12.3%)	24.86 (5.5)		24.86 (5.5)		15.86 (4.5)	
	pT3b	14 (24.6%)	56.06 (12.1)		56.06 (12.1)		33.11 (7.7)	
	pT4a	19 (33.3%	25.89 (5.2)		27.65 (5.6)		17.28 (3.9)	
	pT4b	12 (21.1%)	30.70 (8.3)		29.22 (7.5)		30.97 (8.3)	

**Supplementary Table 12**		*Overall n (%)*	**Mean overall survival (SE) [months]**	*p-value*	**Mean disease specific survival (SE) [months]**	*p-value*	**Mean disease free survival (SE) [months]**	*p-value*
revised pT classification				***<0.001***		***<0.001***		***<0.001***
	pT1	10 (2.2%)	47.12 (13.7)		88.20 (11.4)		88.20 (11.4)	
	pT2	58 (13.0%)	56.89 (5.0)		79.39 (5.0)		75.93 (5.3)	
	pT3a	96 (21.5%)	57.45 (5.2)		81.27 (6.5)		77.94 (6.5)	
	pT3b	144 (32.2%)	36.23 (3.0)		40.79 (3.4)		37.90 (3.5)	
	pT4a	74 (16.6%)	35.72 (4.5)		38.25 (4.7)		33.42 (4.2)	
	pT4b	65 (14.5%)	24.46 (3.9)		28.06 (4.6)		28.93 (4.5)	
WHO grade: low-grade subcohort				**<0.001**		***<0.001***		***<0.001***
	pT1	10 (3.0%)	47.12 (13.7)		88.20 (16.2)		88.20 (16.2)	
	pT2	51 (15.1%)	57.58 (5.4)		75.85 (5.6)		73.94 (5.7)	
	pT3a	82 (24.3%)	58.95 (5.6)		81.40 (6.8)		78.41 (6.8)	
	pT3b	113 (33.5%)	36.22 (3.4)		41.40 (4.0)		38.00 (4.0)	
	pT4a	42 (12.5%)	34.10 (5.8)		35.08 (5.8)		32.86 (5.2)	
	pT4b	39 (11.6%)	28.75 (5.6)		32.20 (6.0)		33.46 (6.1)	
WHO grade: high-grade subcohort				**0.005**		***<0.001***		***0.001***
	pT1	0 (0.0%)	-		-		-	
	pT2	7 (6.4%)	50.43 (9.9)		120.00 (0.0)		120.00 (0.0)	
	pT3a	14 (12.7%)	39.10 (4.6)		45.55 (3.8)		42.63 (4.3)	
	pT3b	31 (28.2%)	34.65 (5.7)		39.13 (6.4)		35.96 (6.6)	
	pT4a	32 (29.1%)	37.06 (6.9)		41.61 (7.3)		34.24 (6.9)	
	pT4b	26 (23.6%)	18.42 (4.8)		20.47 (5.4)		21.51 (5.4)	
pN0 subcohort				***0.024***		***<0.001***		***<0.001***
	pT1	8 (3.6%)	30.41 (8.3)		52.33 (6.3)		52.33 (6.3)	
	pT2	49 (21.8%)	57.95 (5.5)		79.00 (5.4)		77.0 (5.6)	
	pT3a	61 (27.1%)	53.65 (6.1)		81.68 (7.0)		77.42 (7.0)	
	pT3b	64 (28.4%)	44.74 (4.7)		52.43 (5.6)		53.03 (6.0)	
	pT4a	27 (12.0%)	45.39 (8.3)		47.32 (8.1)		37.64 (7.7)	
	pT4b	16 (7.1%)	21.38 (7.6)		26.08 (8.6)		33.27 (8.8)	
pN1/2 subcohort				***<0.001***		***<0.001***		***<0.001***
	pT1	2 (0.9%)	82.00 (19.0)		101.00 (0.0)		101.00 (0.0)	
	pT2	9 (4.1%)	48.75 (11.7)		54.33 (9.9)		54.33 (9.9)	
	pT3a	35 (15.8%)	64.71 (9.2)		74.09 (9.7)		70.33 (10.3)	
	pT3b	80 (36.0%)	29.96 (3.5)		31.75 (3.7)		26.04 (3.4)	
	pT4a	47 (21.2%)	29.87 (4.9)		32.44 (5.2)		30.16 (4.7)	
	pT4b	49 (22.1%)	25.23 (4.3)		27.92 (5.0)		27.48 (5.1)	
pM0				***<0.001***		***<0.001***		***<0.001***
	pT1	10 (2.9%)	47.12 (13.7)		88.20 (11.4)		88.20 (11.4)	
	pT2	57 (16.8%)	56.76 (5.0)		79.25 (5.0)		77.24 (5.2)	
	pT3a	88 (25.9%)	61.35 (5.7)		90.31 (6.8)		86.56 (6.7)	
	pT3b	108 (31.8%)	42.08 (3.7)		49.23 (4.4)		49.18 (4.6)	
	pT4a	41 (12.1%)	46.15 (6.6)		47.40 (6.5)		40.55 (6.3)	
	pT4b	36 (10.6%)	27.17 (5.6)		34.34 (7.2)		35.31 (7.3)	
pM1				***0.923***		***0.886***		***0.069***
	pT1	0 (0.0%)	-		-		-	
	pT2	1 (0.9%)	12.00 (0.0)		12.00 (0.0)		1.00 (0.00)	
	pT3a	8 (7.5%)	28.38 (8.6)		28.38 (8.6)		26.88 (8.4)	
	pT3b	36 (33.6%)	21.72 (3.3)		21.72 (3.3)		16.56 (2.7)	
	pT4a	33 (30.8%)	24.34 (5.3)		27.70 (5.9)		25.80 (5.2)	
	pT4b	29 (27.1%)	20.42 (4.9)		21.15 (5.1)		21.67 (4.7)	

Supplementary Table 13	HR (DSS)	lower CI (95%)	upper CI (95%)	*p-value*
**revised pT classification**				**<0.001**
pT1	1.00			
pT2	2.61	0.92	7.45	
pT3a	2.07	0.72	5.87	
pT3b	5.25	1.90	14.50	
pT4a	6.46	2.30	18.15	
pT4b	9.33	3.26	26.69	
**Gender**				***0.345***
male	1.00			
female	0.89	0.7	1.14	
**WHO-grade**				**0.058**
Low grade	1.00			
High grade	1.28	0.99	1.64	
**pN**				**<0.001**
pN0	1.00			
pN1	1.33	0.98	1.81	
pN2	1.96	1.38	2.78	
**pM**				***<0.001***
pM0	1.00			
pM1	2.56	1.89	3.46	
**Resection margin**				**0.019**
R0	1.00			
R1	1.56	1.02	2.39	
R2	1.74	1.09	2.76	
**Age group**				**0.001**
Below median	1.00			
Median and above	1.54	1.21	1.96	

Supplementary Table 14		HR (DSS)	lower CI (95%)	upper CI (95%)	*p-value*
**revised pT classification**					**0.008**
	pT1	1.00			
	pT2	1.28	0.40	4.06	
	pT3a	1.12	0.36	3.53	
	pT3b	2.29	0.77	6.80	
	pT4a	2.56	0.80	8.21	
	pT4b	6.76	1.63	28.03	
**Gender**					***0.586***
	male	1.00			
	female	0.89	0.57	1.37	
**WHO-grade**					**0.322**
	Low grade	1.00			
	High grade	1.26	0.80	1.97	
**pN**					**<0.001**
	pN0	1.00			
	pN1	1.57	0.88	2.78	
	pN2	4.71	2.70	8.22	
**pM**					***0.002***
	pM0	1.00			
	pM1	2.59	1.43	4.68	
**Resection margin**					**0.636**
	R0	1.00			
	R1	0.97	0.41	2.30	
	R2	1.64	0.21	12.63	
**Age group**					**<0.001**
	Below median	1.00			
	Median and above	2.28	1.44	3.62	

Supplementary Table 15	HR (DSS)	lower CI (95%)	upper CI (95%)	*p-value*
**revised pT classification**				**<0.001**
pT1	1.00			
pT2	1.72	0.22	13.65	
pT3a	2.24	0.30	16.67	
pT3b	6.15	0.85	44.57	
pT4a	6.15	0.83	45.44	
pT4b	8.62	1.17	63.49	
**Gender**				***0.500***
female	1.00			
male	0.91	0.68	1.20	
**WHO-grade**				**0.857**
Low grade	1.00			
High grade	1.03	0.75	1.43	
**pN**				**0.195**
pN0	1.00			
pN1	1.19	0.82	1.72	
pN2	1.29	0.88	1.90	
**pM**				***<0.001***
pM0	1.00			
pM1	1.95	1.34	2.85	
**Resection margin**				**0.226**
R0	1.00			
R1	1.22	0.72	2.06	
R2	1.35	0.83	2.17	
**Age group**				**0.002**
Below median	1.00			
Median and above	1.59	1.19	2.12	

Supplementary Table 16

		pT3 conventional measurement		Number of cases	Kappa-value	p-value
		pT3a	pT3b			
pT3 digital measurement	pT3a	74	1	75		
	pT3b	5	45	50		
Number of cases		79	46	125	0.89	<0.001

Supplementary material legends

Supplementary Figure 1: Disease specific survival analysis of pT3a vs. pT3b within all pT3 colon cancers (A) and in all pT3 rectal cancers (B), with a comparable survival impact in both anatomic subgroups, that is also visible in comparison to other pT stages, as separately shown for colon cancers (C) and rectal cancers (D).

Supplementary Table 1: Distribution and prognostic relevance of basic clinicopathological and morphological parameters in the training cohort (Munich).

Supplementary Table 2: Distribution and prognostic relevance of basic clinicopathological and morphological parameters in validation cohort 1 (Mainz).

Supplementary Table 3: Distribution and prognostic relevance of basic clinicopathological and morphological parameters in validation cohort 2 (Bayreuth).

Supplementary Table 4: Impact on survival parameters (log-rank test) of the proposed pT3a/b substratification in pT3 CRC subgroup of the training cohort (Munich), including pN, pM and WHO-grade subgroups.

Supplementary Table 5: Impact on survival parameters (log-rank test) of the proposed pT3a/b substratification in pT3 CRC subgroup of validation cohort 1 (Mainz), including pN, pM and WHO-grade subgroups.

Supplementary Table 6: Impact on survival parameters (log-rank test) of the proposed pT3a/b substratification in pT3 CRC subgroup of validation cohort 2 (Bayreuth), including pN, pM and WHO-grade subgroups.

Supplementary Table 7: Multivariate analysis for Disease specific survival (DSS) of the proposed pT3a/b substratification (Cox-regression) in the pT3 CRC subgroup of the training cohort (Munich) including age, gender, WHO-grade, pN, pM and resection margin.

Supplementary Table 8: Multivariate analysis for Disease specific survival (DSS) of the proposed pT3a/b substratification (Cox-regression) in the pT3 CRC subgroup of validation cohort 1 (Mainz), including age, gender, WHO-grade, pN, pM and resection margin.

Supplementary Table 9: Multivariate analysis for Disease specific survival (DSS) of the proposed pT3a/b substratification (Cox-regression) in the pT3 CRC subgroup of validation cohort 2 (Bayreuth), including age, gender, WHO-grade, pN, pM and resection margin.

Supplementary Table 10: Impact on survival parameters (log-rank test) in all CRCs of the revised pT classification including all pT-stages and the proposed pT3a/b substratification in the training cohort (Munich), including pN, pM and WHO-grade subgroups.

Supplementary Table 11: Impact on survival parameters (log-rank test) in all CRCs of the revised pT classification including all pT-stages and the proposed pT3a/b

substratification in validation cohort 1 (Mainz), including pN, pM and WHO-grade subgroups.

Supplementary Table 12: Impact on survival parameters (log-rank test) in all CRCs of the revised pT classification including all pT-stages and the proposed pT3a/b substratification in validation cohort 2 (Bayreuth), including pN, pM and WHO-grade subgroups.

Supplementary Table 13: Multivariate analysis for Disease specific survival (DSS) in all CRCs of the revised pT classification including all pT-stages and the proposed pT3a/b substratification of the training cohort (Munich) including age, gender, WHO-grade, pN, pM and resection margin.

Supplementary Table 14: Multivariate analysis for Disease specific survival (DSS) in all CRCs of the revised pT classification including all pT-stages and the proposed pT3a/b substratification of validation cohort 1 (Mainz), including age, gender, WHO-grade, pN, pM and resection margin.

Supplementary Table 15: Multivariate analysis for Disease specific survival (DSS) in all CRCs of the revised pT classification including all pT-stages and the proposed pT3a/b substratification of validation cohort 2 (Bayreuth), including age, gender, WHO-grade, pN, pM and resection margin.

Supplementary Table 16: Concordance of digital measurements versus non-digital measurements using a conventional light-microscope and a ruler.
